# Generalized Portrait of Cancer Metabolic Pathways Inferred from a List of Genes Overexpressed in Cancer

**DOI:** 10.1155/2014/646193

**Published:** 2014-08-27

**Authors:** Eugenia Poliakov, David Managadze, Igor B. Rogozin

**Affiliations:** ^1^Laboratory of Retinal Cell and Molecular Biology, National Eye Institute, National Institutes of Health, Bethesda, MD 20892, USA; ^2^National Center for Biotechnology Information, National Library of Medicine, National Institutes of Health, Bethesda, MD 20894, USA

## Abstract

More than half a century from postulated Warburg theory of cancer cells origin, a question of changed metabolism in cancer is again taking the central place. Generalized picture of cancer metabolism was replaced by analysis of signaling and oncogenes in each type of cancer for several decades. However, now empowered with wealth of knowledge about tumor suppressors, oncogenes, and signaling pathways, reprogramming of cellular metabolism (e.g., increased glycolysis to respiration ratio in cancer cells) reemerged as an important element of cancer progression. To analyze level of expression of various proteins including metabolic enzymes across various cancers we used dbEST and Unigene data. We delineated a list of genes that are overexpressed in different types of cancer. We also grouped overexpressed enzymes into KEGG pathways and analyzed adjacent pathways to describe enzymatic reactions that take place in cancer cells and to identify major players that are abundant in cancer protein machinery. Glycolysis/gluconeogenesis and oxidative phosphorylation are the most abundant pathways although several other pathways are enriched in genes from our list. Ubiquitously overexpressed genes could be marked as nonspecific cancer-associated genes when analyzing genes that are overexpressed in certain types of cancer. Thus the list of overexpressed genes may be a useful tool for cancer research.

## 1. Introduction

More than half a century from postulated Warburg theory of cancer cells origin [1], question of changed metabolism in cancer is again taking central place. For several decades a generalized picture of cancer metabolism was replaced by analysis of signaling and oncogenes in each type of cancer. However, now empowered with wealth of knowledge about tumor suppressors, oncogenes, and signaling pathways, reprogramming of cellular metabolism first described as Warburg effect (increased glycolysis to respiration ratio in cancer cells) [2] reemerged as important element of cancer progression [3–5]. The metabolism of one molecule of glucose to two molecules of pyruvate in glycolysis has a net yield of two molecules of ATP. Glycolysis does not require or consume oxygen. Eukaryotic aerobic respiration (oxidative phosphorylation) produces approximately 34 additional molecules of ATP for each glucose molecule. The lower-energy production, per glucose, of anaerobic respiration relative to aerobic respiration, results in greater flux through the pathway under hypoxic (low-oxygen) conditions. It has been hypothesized that, in these cells, glycolytic enzymes associate into a large complex, which results in an increased efficiency of glycolytic flux [6]. Another explanation is based on alternative glycolytic pathway that bypasses ATP production but produces pyruvate [7]. Recent analysis of microarray data across major cancer types showed activation of certain metabolic pathways in cancer cells [8, 9]. These data confirmed that cancer cells upregulate biosynthesis and metabolism of certain nutrients like glycine and glutamine along with upregulated glycolysis [10, 11].

Analysis of cancer molecular signatures deduced from genomics data recently appeared in the literature [12–14]. For example, overexpressed membrane receptors as suggested by an analysis of ESTs (expressed sequences tags) could be used as hallmark of cancer cells [15]. EST datasets were also analyzed by Aouacheria and coworkers to distinguish between normal and tumor tissues [16]. Analysis was done in a tissue-specific manner but demonstrated similar patterns of enzymes enrichment in various cancers, for example, glycolytic enzymes, alpha enolase ENO1 (EC 4.2.1.11), pyruvate kinase, muscle PKM2 (EC 2.7.1.40), and glyceraldehyde-3-phosphate dehydrogenase GAPDH (EC 1.2.1.12), were found in three different types of cancer [16]. This result demonstrates that some genes are ubiquitously highly expressed in cancer cells and such genes may be important hallmarks of cancer cells [12].

To analyze level of expression of various proteins including metabolic enzymes across various cancers we used EST data. We also used the NCBI Unigene data across the panel of cancers. We grouped overexpressed enzymes into KEGG pathways and manually analyzed adjacent pathways to analyze enzymatic reactions that take place in cancer cells and to identify major players that are abundant in cancer protein machinery.

## 2. Materials and Methods

The 23,586 nonredundant coding sequences (CDS) of human genes from the human genome draft build 35, the April 2012 freeze, obtained at the NCBI ftp server were used as reference sequences to be compared with the EST sequences. The EST sequences were from the dbEST release of June 2012. Each EST library was assigned to either cancer-related (1,437 libraries; 1,848,538 ESTs (26%)) or normal tissue (7,309 libraries; 5,276,385 ESTs (74%)) bins (the file EST_libraries.xls is available at ftp://ftp.ncbi.nlm.nih.gov/pub/managdav/paper_suppl/est_cancer_pathways/). The CDS set was searched against dbEST using the BLASTN program with the default parameters. The number of ESTs with at least 97% identity and alignment length of at least 200 nucleotides or longer was counted. The Unigene database was used to verify results of BLASTN-dbEST searches.

We used two statistical filters to delineate a set of genes overexpressed in cancer (10 genes with the largest number of cancer EST libraries are shown in the Table 1).

1. We included a gene in a list of preliminarily candidates if the number of cancer ESTs is greater in comparison with normal ESTs using Fisher 2 × 2 exact test (*X*1-*X*2,* X*2,* X*3-*X*4,* X*4) (an arbitrary threshold *P* value = 0.01).* X*1 is the total number of cancer ESTs,* X*2 is the number of cancer ESTs for a given gene,* X*3 is the total number of normal ESTs, and* X*4 is the number of normal ESTs for a given gene.
2. We accepted a gene in the final list (Supplementary Table S1; Supplementary Material available online at http://dx.doi.org/10.1155/2014/646193) from the preliminarily candidates if the number of cancer ESTs per EST library is greater in comparison with normal ESTs per EST library using Fisher 2 × 2 exact test (*X*1,* X*2,* X*3,* X*4) (an arbitrary threshold *P* value = 0.01).* X*1 is the total number of cancer ESTs for a given gene,* X*2 is the number of cancer EST libraries with at least one BLASTN hit for a given gene,* X*3 is the total number of normal ESTs for a given gene, and* X*4 is the number of normal EST libraries with at least one BLASTN hit for a given gene. In other words, if a gene has an EST-detectable expression level in an EST library, it should be expressed in cancer EST libraries higher than in normal EST libraries.

A KEGG pathway enrichment in genes overexpressed in cancer libraries (Table 2) was estimated using the Fisher 2 × 2 exact test (*X*1-*X*2,* X*2,* X*3-*X*4,* X*4) and the KEGG database [17].* X*1 is the total number of proteins in the initial set,* X*2 is the number of proteins in a given pathway,* X*3 is the total number of genes that are overexpressed in cancer,* X*4 is the total number of genes in a given pathway that are overexpressed in cancer. It should be noted that all three statistical tests are deliberately simplified because EST datasets can be used as semiquantitative estimators of gene expression and are considered to be an approximate measure of expression in this study (see Section 4).

## 3. Results

### 3.1. Analysis of Genes/Pathways Overexpressed in Cancer

We analyzed large collections of EST libraries associated with cancer and compared them to EST libraries from normal tissues. We tried to select genes that are highly expressed in cancer and the level of expression is substantially different between cancer and normal samples taking into account that EST data is inherently semiquantitative. We used two statistical filters to delineate a list of genes that are highly expressed in many cancer-associated EST libraries (see Section 2). In this way we attempted to remove genes that are associated with specific types of cancer in order to reconstruct a generalized portrait of a cancer cell. We delineated 394 genes that are overexpressed in cancer cells according to statistical filters (see Section 2, Supplementary Table S1; the file Supp_table_S1.xls is also available at ftp://ftp.ncbi.nlm.nih.gov/pub/managdav/paper_suppl/est_cancer_pathways/). A wide variety of functional themes were found in this list and many products of these genes are proteins involved in translation. For example, among 10 genes with the largest number of cancer-related EST libraries, two ribosomal proteins and two translation elongation factors were found (Table 1). This is consistent with many previous observations of highly significant elevation of protein synthesis rates and the expression of several translation components in various cancer cells indicating an importance of ribosome function and translational control in tumor progression [18–21].

We used the KEGG database [17] to assign proteins overexpressed in cancer to various metabolic pathways. We found several KEGG pathways that are significantly enriched in proteins overexpressed in cancer whereas many other pathways do not show a significant enrichment in genes overexpressed in cancer although they contain two or more genes overexpressed in cancer (Table 2). We discuss all these pathways below in more detail.

### 3.2. Glycolysis/Gluconeogenesis

Ten enzymes (TPI1, PGAM1, ENO1, PKM2, ALDH3A1, GAPDH, LDHB, ALDH3B1, ALDH3B2, and ALDOA) from the glycolysis pathway (62 genes) are overexpressed in cancer (Table 2 and Supplementary Table S1). The PKM2 embryonic isoform of pyruvate kinase is important for cancer metabolism and tumor growth [22, 23]. PGAM1 and PKM2 are involved in the alternative glycolytic pathway producing pyruvate [7]. Our data confirm that glycolysis is the central metabolic process for cancer cells.

### 3.3. Oxidative Phosphorylation

Ten enzymes (ATP5F1, SDHD, ATP5B, UQCRC1, NDUFA7, ATP6V0E1, NDUFB9, NDUFS2, NDUFB5, and CYC1) out of 135 proteins participating in this pathway were overexpressed in cancer (Table 2 and Supplementary Table S1). It was demonstrated that hypoxic cancer cells maintain active, though diminished, oxidative phosphorylation even at 1% oxygen. ATP production in these cells is around 40% of ATP production under normal oxygen conditions and their results suggest that, under hypoxia, the autophagy is required to support ATP production [24]. Our data demonstrate that the oxidative phosphorylation is important for cancer survival and growth. Overexpression of LDHB according to cancer EST analysis (Supplementary Table S1) is also pointing to a possible utilization of lactate for the oxidative metabolism [25, 26].

### 3.4. Pyruvate Metabolism

Pyruvate is produced in glycolysis and is used for lactate production. High ratio of lactate/pyruvate is used for metabolic imaging of prostate cancer [27]. Lactate is a prominent substrate that fuels the oxidative metabolism of oxygenated tumor cells. There is a symbiosis in which glycolytic and oxidative tumor cells mutually regulate their access to energy metabolites [25]. Preferential utilization of lactate for oxidative metabolism spares glucose that may in turn reach hypoxic tumor cells [26]. Five enzymes (LDHB, MDH2, PKM2, AKR1B1, and AKR1B10) out of 40 enzymes in pyruvate metabolism pathway are overexpressed in cancer (Table 2 and Supplementary Table S1). Surprisingly, we found an overexpression of the lactate dehydrogenase B enzyme in certain cancers (Supplementary Table S1) and no significant upregulation of the lactate dehydrogenase A enzyme that has been suggested to have a ubiquitous role in tumor metabolism and growth [28].

### 3.5. Metabolism of Aldehydes (Xenobiotics, Drugs) by Cytochrome P450

We found that our EST dataset is enriched in various aldehyde dehydrogenases (ALDH3A1, ALDH3B1, and ALDH3B2) (Table 2 and Supplementary Table S1). Aldehyde dehydrogenase superfamily plays an important role in the enzymatic detoxification of endogenous and exogenous aldehydes and in the formation of molecules that are important in cellular processes. Additionally, ALDH3B1 expression is upregulated in many human tumors and this enzyme is catalytically active toward aldehydes derived from lipid peroxidation, suggesting a potential role against oxidative stress [29]. Moreover, three members of aldo-keto reductases (AKRs), AKR1C1, AKR1C2, and AKR1C3, are overexpressed in cancer (Table 2 and Supplementary Table S1). The resistance towards the chemotherapeutic drug cisplatin in colon cancers is believed to be a result of decreased sensitivity toward cellular damages evoked by oxidative stress-derived aldehydes, 4-hydroxy-2-nonenal and 4-oxo-2-nonenal, that are detoxified by AKR1C1 and AKR1C3 [30]. Metabolism of xenobiotics by cytochrome P450 pathway has six overexpressed enzymes from total 70 enzymes. Furthermore, one member of the superfamily of short-chain dehydrogenases/reductases (SDR) was found to be overexpressed in cancer (DHRS2 or Hep27, Supplementary Table S1), SDR catalyzes the NADPH-dependent reduction of dicarbonyl compounds [31].

### 3.6. Fructose and Mannose Metabolism

Fructose provides an alternative carbon source for glycolysis, entering downstream of glucose and bypassing two key rate-limiting steps. Whereas glucose favors overall growth kinetics, fructose enhances protein and nucleotide synthesis and appears to promote a more aggressive cancer phenotype [32, 33]. Four enzymes (TPI1, AKR1B10, ALDOA, and AKR1B1) out of 34 enzymes in this pathway are overexpressed in cancer (Table 2 and Supplementary Table S1). All these enzymes participate in a variety of metabolic pathways.

### 3.7. One Carbon Pool by Folate

Three enzymes (MTHFD2, ATIC, and SHMT2) in this pathway were found to be overexpressed in many cancers (Table 2). This result suggests that most of tetrahydrofolate (THF) forms (5,6,7,8-THF; 10-formyl-THF; 5,10-methenyl-THF; 5,10-methylene-THF) are synthesized in cancer cells. Byproduct of serine hydroxymethyltransferase 2 (SHMT2) is glycine and its biosynthesis and metabolism is upregulated in proliferating cancer cells [11]. The 5-methyltetrahydrofolate-homocysteine methyltransferase (MTR), which synthesizes L-methionine and THF from L-homocysteine and 5-methyltetrahydrofolate, was not included in our list (Supplementary Table S1). However, the MTR cofactor vitamin B12 (cobalamin) ATP-transporter MMADHC is overexpressed in cancer (Supplementary Table S1).

### 3.8. TCA Cycle (Citrate Cycle)

Three enzymes (FH, SDHD, and MDH2) were found to be overexpressed in the TCA cycle (32 enzymes, Table 2 and Supplementary Table S1). Even though mutations in FH and SDHD lead to development of tumors [34], overexpression of these three enzymes is likely to lead to accumulation of oxaloacetate. TCA cycle is subject to metabolic reprogramming in cancer cells [35, 36]. In the transformed cells, the tricarboxylic acid (TCA) cycle was active but was characterized by an efflux of substrates for use in biosynthetic pathways, particularly fatty acid synthesis. Glutamine metabolism in these cells supports restoration of oxaloacetate for continued TCA cycle function as well as NAPDH production [37]. We found in our list of genes (Supplementary Table S1) the glutamine transporter SLC1A5 that is important for survival of lung cancer cells [38].

### 3.9. Glycerolipid Metabolism

We observed overexpression of monoglyceride lipase (MGLL) that catalyzes the conversion of monoacylglycerides to free fatty acids and glycerol. Expression of this gene may play a role in tumorigenesis and metastasis. Also aldo-keto reductases AKR1B1 and AKR1B10 enriched in cancer ESTs catalyze reduction of D-glyceraldehyde to glycerol and 2-hydroxypropanal to propane-1,2-diol in glycerolipid metabolism. A total of three enzymes out of 49 enzymes in this pathway are overexpressed in cancer although the pathway enrichment is not significant (Table 2 and Supplementary Table S1).

### 3.10. Steroid Hormone Biosynthesis

The same three members of AKRs (AKR1C1, AKR1C2, and AKR1C3) that are involved in metabolism of aldehydes (xenobiotics, drugs) by cytochrome P450 and are overexpressed in cancer (see above) participate in the steroid biosynthesis pathway and could play more than detoxifying role in cancer cells. Only these three enzymes are present in our list among 55 enzymes in this pathway (Table 2).

### 3.11. Pentose Phosphate Pathway

Two enzymes (ALDOA, PGD) from pentose phosphate pathway (27 genes) are overexpressed in cancer (Table 2 and Supplementary Table S1). This pathway is adjacent to glycolysis pathway and feeds into purine and pyrimidine metabolism producing 5-phospho-α-D-ribosyl 1-pyrophosphate (PRPP).

### 3.12. Purine Metabolism/Purine* De Novo* Biosynthesis

The* de novo* synthesis of the purine ring is mostly required in cells when DNA replication occurs and the activity of the metabolic pathway in most of tissues is relatively low [39]. Differentiated cells largely employ the salvage pathway, which recycle nucleotides by retrieving the purine ring after nucleic acid or coenzyme breakdown [39].

Four enzymes in the purine metabolism/purine* de novo* biosynthesis KEGG pathway are overexpressed in cancer (Table 2 and Supplementary Table S1). ATIC encodes a bifunctional protein that catalyzes the last two steps of the* de novo* purine biosynthetic pathway. ATIC inhibitors are being developed as anticancer therapy [40, 41]. IMPDH2 encodes the rate-limiting enzyme in the* de novo* guanine nucleotide biosynthesis. These two enzymes are key enzymes in the* de novo* synthesis of purine nucleotides. The other two enzymes involved in this pathway are not exclusive for this pathway. PKM2 catalyzes the transfer of a phosphoryl group from phosphoenolpyruvate to ADP (GDP), generating ATP (GTP) and pyruvate. The DNA polymerase delta subunit 2 (POLD2) is involved in DNA synthesis and repair [42].

### 3.13. Pyrimidine Metabolism/Pyrimidine* De Novo* Biosynthesis

Carbamoyl-phosphate synthetase 2, aspartate transcarbamylase, and dihydroorotase (CAD) enzyme catalyzing the first three steps in the 6-step pathway of pyrimidine* de novo* biosynthesis are overexpressed in cancer (Supplementary Table S1). Additionally, the uridine phosphorylase 1 (UPP1) participates in degradation and salvage of pyrimidine ribonucleosides. The DNA polymerase delta subunit 2 (POLD2) incorporates pyrimidine and purine nucleotides in DNA. Three enzymes in this KEGG pathway (98 genes) are overexpressed in cancer (Table 2 and Supplementary Table S1).

### 3.14. Cysteine and Methionine Metabolism

Adenosylhomocysteinase (AHCY) enzyme that produces L-homocysteine and adenosine by hydrolysis of S-adenosyl-L-homocysteine was found to be overexpressed in cancer (Table 2). This protein is in cysteine and methionine metabolism pathway and also may be used by the MTR enzyme in the one carbon pool by folate pathway (see above). LDHB is another enzyme in the pathway that is overexpressed in cancer (Supplementary Table S1).

### 3.15. Aminoacyl tRNA Biosynthesis

The cytoplasmic methionyl-tRNA synthetase (MARS) and the cytoplasmic alanyl-tRNA synthetase (AARS) enzymes that charge tRNAs with their cognate amino acids were found to be overexpressed in cancer (Table 2 and Supplementary Table S1).

### 3.16. Fatty Acid Metabolism

Two enzymes that participate in the fatty acid metabolism are overexpressed in cancer. The mitochondrial enoyl CoA hydratase, short chain 1 (ECHS1) catalyzes the second step of the mitochondrial fatty acid beta-oxidation pathway. Second enzyme is the stearoyl-CoA desaturase (delta-9-desaturase) (SCD), which is involved in synthesis of monounsaturated fatty acids, mostly the oleic acid. Recently, it has been shown that cancer survival is dependent on unsaturated fatty acids and is implicated SCD in this process [43]. Additionally, SCD inhibition causes cancer cell death by depleting monounsaturated fatty acids [44]. We also found that the fatty acid transporter SCP2, which protects fatty acids from oxidation, is overexpressed in cancer. SCD expression is upregulated by retinoic acid in various untransformed cell lines [45]. In our EST database we see an overexpression of cellular retinoic acid binding protein 2 (CRABP2) together with MYCN proteins that have been shown to be upregulated and correlated in variety of cancers [46]. Aldo-keto reductases overexpressed in our cancer dataset (AKR1C1, AKR1B10, and AKR1C3) are involved in the reduction of retinal to retinol (Table 2 and Supplementary Table S1).

### 3.17. N-Glycan Biosynthesis

Glycosylation is one of the most common posttranslational modification reactions and changes in oligosaccharide structures are associated with many physiological and pathological events, including cell growth, migration, differentiation, and tumor invasion [47]. N-Glycans are involved in cancer progression and MGAT4 mainly participate in branching of N-glycans [48]. Number of Mgat4b transcripts increased considerably in diethylnitrosamine-induced hepatocellular carcinoma mice [49]. DPM1 is enzyme that forms dolichol phosphate mannose (Dol-P-Man), which is the mannosyl donor in pathways leading to N-glycosylation and O-mannosylation. We found these two enzymes to be overexpressed in cancer (2 out of 46 enzymes in this pathway). One enzyme (farnesyl diphosphate synthase FDPS) from the terpenoid biosynthesis pathway (1 out of 15 enzymes) that feeds in N-glycan biosynthesis pathway was also overexpressed in cancer (Table 2 and Supplementary Table S1).

### 3.18. Phenylalanine/Tyrosine/Histidine Metabolism

Three enzymes (ALDH3B1, ALDH3B2, and ALDH3A1) that participate in the oxidation of acetaldehyde to acetate in the glycolysis/gluconeogenesis pathway and are involved in xenobiotics and drug oxidation by cytochrome P450 also participate in metabolism of amino acids phenylalanine (18 enzymes), tyrosine (42 enzymes), and histidine (29 enzymes) (Table 2 and Supplementary Table S1). We did not attempt to analyze enrichment of phenylalanine/tyrosine/histidine metabolism due to the obvious overlap with the glycolysis/gluconeogenesis pathway that is highly enriched in cancer-associated genes (Table 2).

### 3.19. Enzymes Introducing Posttranslational Modifications

Introduction of disulfide bonds by Cys mutations has been shown to improve the physical stability of some proteins [50]. We observed that three disulfide isomerases, AGR2, AGR3, and TXNDC5, are overexpressed in cancer (Table 2). AGR2 is the prooncogenic protein that could be used as a tumor biomarker [51]. AGR3 is overexpressed by a hormone- (estrogen-receptor α-) independent mechanism and identifies a novel protein-folding associated pathway that could mediate resistance to DNA-damaging agents in human cancers [52].

We also observed that one atypical excreted disulfide oxidase quiescin sulfhydryl oxidase 1 (QSOX1), which supports cell-matrix adhesion and cell migration [53], is markedly overexpressed in cancer (Supplementary Table S1).

Third class of protein modifying enzymes we observed in our dataset (Supplementary Table S1) is peptidyl-prolyl isomerases (PPIA) that are known to accelerate protein folding. PPIA is one member we found in our list (Supplementary Table S1). Another member is the peptidyl-prolyl isomerase FKBP4 that is a cochaperone which activates RNA interference-mediated silencing in mammalian cells [54, 55].

### 3.20. Additional Antioxidant Systems

Our list of genes overexpressed in cancer is also enriched in certain antioxidant enzymes (peroxiredoxins 1 and 3 (PRXD1 and PRXD3) and cytochrome b561 (CYB561)) (Supplementary Table S1). Peroxiredoxins reduce hydrogen peroxide and alkyl hydroperoxides and CYB561 is involved in a reduction of ascorbate radicals.

## 4. Discussion

The use of EST to measure gene expression requires a lot of caution because many libraries may have insufficient coverage of low and moderately expressed genes. It should be noted that EST-based expression analyses were used in several studies of cancer cells [15, 16]. We assume that cancer ESTs are a good tool to study general properties of gene highly expressed in cancer cells because the statistics were collected over a large number of EST libraries that compensate to some extent a semiquantitative nature of any EST-based expression measure. We have no doubt that when RNAseq data will be available for a range of libraries/tissues similar to dbEST, this will substantially improve the generalized portrait of cancer metabolic pathways.

There are many computational approaches for analysis of metabolic and signaling pathway enrichment [56]. We used the simplest approach in this paper: the Fisher exact test (Table 2). The DAVID system [57] uses a more conservative implementation of this test. We analyzed the dataset using the DAVID system and found a very similar list of metabolic pathways although only three pathways were significantly enriched in genes that were found to be overexpressed in cancer (Supplementary Table S1): glycolysis/gluconeogenesis (EASE Score = 1.2 × 10^−4^), oxidative phosphorylation (EASE Score = 2.6 × 10^−2^), and metabolism of xenobiotics by cytochrome P450 (EASE Score = 4.5 × 10^−2^). We do not see this as a contradiction with our results (Table 2) and differences in *P* values are likely to reflect known methodological problems with the analysis of pathway enrichment [56]. We also observed obvious problems with some KEGG pathways: for example, for the purine metabolism, the* de novo* purine biosynthesis pathway and the salvage pathway are merged into one KEGG pathway (Table 2). However it was shown that only* de novo* purine biosynthesis is overexpressed in cancer cells [40, 41]. The same problem was found for the pyrimidine metabolism (Table 2). Therefore the insignificant *P* values for the pathway enrichment (Table 2) do not necessarily indicate that such pathways are not important for cancer initiation and progression.

There are numerous attempts to build a census of human cancer genes [12–14, 58, 59]; for example, Futreal et al. [58] and Santarius et al. [12] identified ~400 candidate cancer-related genes. An important direction along this venue of research is development of predictive models for cancer-associated genes that could accelerate their identification. Such models have been developed for specific types of cancer. One example of such studies is a complex statistical model for the prediction of prostate cancer genes [60]. In our study we applied a complementary approach using simplified statistical filters for prediction of genes that are overexpressed in all available cancer EST libraries without classifying them into types and subtypes (Table 1 and Supplementary Table S1). In other words, we tried to delineate a list of broadly overexpressed genes and a generalized portrait of cancer metabolic pathways that are expected to be overrepresented in this list. Another important difference is that in this list we do not expect that genes involved in metabolic pathways have many somatic nonsynonymous mutations that are likely to cause inactivation or gain of a new function; an excess of such mutations has been used in previous attempts to build a census human cancer genes [12–14, 58, 59]. Genes that are found in many cancer EST libraries (Supplementary Table S1) should be marked (or even removed) as nonspecific cancer-associated genes when researchers analyze genes overexpressed in certain types of cancer. On the other hand, we know that cancer modifies normal metabolic processes to fulfill its high growth and energy demands and the above described metabolic pathways seem to be universally central for cancerous growth and progression. Enzymes that are highly expressed in cancer along these metabolic pathways could provide multiple targets for desirable inhibition of cancer progression.

## Supplementary Material

A list of genes overrepresented in cancer EST libraries. We used two statistical filters for prediction of genes that are overexpressed accross all available cancer EST libraries without classifying them into types and subtypes. The name, the total number of cancer ESTs, the number of cancer ESTs, the total number of normal ESTs, the number of normal ESTs, and a brief description is provided for each gene.

## Figures and Tables

**Table 1:** Examples of genes overexpressed in cancer: ten genes with the largest number of EST libraries.

Gene	Number of cancer ESTs	Number of cancer EST libraries	Number of normal ESTs	Number of normal EST libraries	Gene description
EEF1A1	26747	380	54872	1436	Translation elongation factor
GAPDH	11388	300	9446	858	Glyceraldehyde-3-phosphate dehydrogenase
ACTG1	9831	295	8826	879	Actin gamma
FTH1	5264	260	6627	758	Ferritin, heavy polypeptide 1
EEF1G	6575	255	7185	783	Eukaryotic translation elongation factor 1 gamma
RPLP0	6583	253	6400	683	Ribosomal protein, large subunit, P0
HSP90AB1	4035	253	7178	801	Heat shock protein 90 kDa alpha (cytosolic), class B member 1
PKM2	4794	248	7593	760	Pyruvate kinase, muscle
FTL	4495	246	7784	599	Ferritin, light polypeptide
RPL3	6278	242	7463	753	Ribosomal protein L3

The complete list of genes overexpressed in cancer is shown in Supplementary Table S1 (also available at ftp://ftp.ncbi.nlm.nih.gov/pub/managdav/paper_suppl/est_cancer_pathways/).

**Table 2:** KEGG metabolic pathways enriched in genes that are overexpressed in cancer.

Pathway	Number of genes in a pathway (*X*2)	Number of genes overexpressed in cancer (*X*4)	Pathway enrichment
Glycolysis/gluconeogenesis	62	10	2.8 × 10^−7^
Oxidative phosphorylation	135	10	0.00015
Pyruvate metabolism	40	5	0.00083
Metabolism of xenobiotics by cytochrome P450	70	6	0.0016
Fructose and mannose metabolism	34	4	0.0034
One carbon pool by folate	17	3	0.0041
TCA cycle (citrate cycle)	32	3	0.02
Glycerolipid metabolism	49	3	0.054
Steroid hormone biosynthesis	55	3	0.07
Pentose phosphate pathway	27	2	0.082
Purine metabolism/purine *de novo* biosynthesis	159	4	0.35
Pyrimidine metabolism/pyrimidine *de novo* biosynthesis	98	3	0.23
Cysteine and methionine metabolism	34	2	0.12
Aminoacyl tRNA biosynthesis	41	2	0.16
Fatty acid metabolism	43	2	0.17
N-Glycan biosynthesis	46	2	0.19
Phenylalanine/tyrosine/histidine metabolism	18/42/29	3	—
Enzymes introducing posttranslational modifications	?	6	—
Additional antioxidant systems	?	3	—

Pathway enrichment is estimated using the Fisher 2 × 2 exact test (*X*1-*X*2, *X*2, *X*3-*X*4, *X*4). *X*1 is the total number of genes in the initial set (23,586 genes), *X*2 is the number of proteins in a given pathway, *X*3 is the total number of genes that are overexpressed in cancer (394 genes), and *X*4 is the total number of genes in a given pathway that are overexpressed in cancer.
